# Juvenile Hormone Biosynthesis in Insects: What Is New, What Do We Know, and What Questions Remain?

**DOI:** 10.1155/2014/967361

**Published:** 2014-10-19

**Authors:** Fernando G. Noriega

**Affiliations:** Department of Biological Sciences, Florida International University, Miami, FL 33199, USA

## Abstract

Our understanding of JH biosynthesis has significantly changed in the last years. In this review I would like to discuss the following topics: (1) the progresses in understanding the JH biosynthesis pathway. Access to genome sequences has facilitated the identification of all the genes encoding biosynthetic enzymes and the completion of comprehensive transcriptional studies, as well as the expression and characterization of recombinant enzymes. Now the existence of different flux directionalites, feed-back loops and pathway branching points in the JH biosynthesis pathways can be explored; (2) the new concepts in the modulation of JH synthesis by allatoregulators. The list of putative JH modulators is increasing. I will discuss their possible role during the different physiological states of the CA; (3) the new theoretical and physiological frameworks for JH synthesis analysis. I will discuss the bases of the flux model for JH biosynthesis. JH plays multiple roles in the control of ovary development in female mosquitoes; therefore, the CA presents different physiological states, where JH synthesis is altered by gating the flux at distinctive points in the pathway; (4) in the final section I will identify new challenges and future directions on JH synthesis research.

## 1. Introduction

Juvenile hormone (JH) regulates development and reproductive maturation in insects [1, 2]; therefore, interruption of JH biosynthesis has been considered as a strategy for the development of target-specific insecticides [3]. Although degradation plays a role, JH titer is primarily determined by the rate of biosynthesis in the* corpora allata* gland (CA). A number of recent reviews have summarized the current knowledge on JH biosynthesis in insects [1, 2], as well as its potential as a target for insecticide discovery [3]. In the present review I would like to focus on the discussion of some new advances in the field and on the identification of outstanding questions that remain to be addressed, as well as the potential directions for future research.

Our understanding of JH biosynthesis has significantly changed over the past few years. Access to genome sequences has facilitated the identification of all the genes encoding JH biosynthetic enzymes [4–6] and the completion of comprehensive transcriptional studies [5, 6], as well as the expression and characterization of recombinant JH biosynthetic enzymes [7–10]. The development of new technologies is facilitating the analysis of JH biosynthesis rates, enzymatic activities, and metabolite pool sizes in the CA [11, 12]. In addition, new theoretical and physiological frameworks are simplifying JH synthesis analysis [12].

This review will emphasize the work that has been done on the biosynthesis of JH III in the mosquito* Aedes aegypti*. The importance of* A. aegypti* as a vector of diseases has attracted the interest of scientists and funding agencies for many years. Consequently, there is ample information available on biological, ecological, anatomical, and physiological aspects of this mosquito, both published in primary research articles and summarized in excellent textbooks [13, 14]. Vectorbase is an excellent web resource available for genomic analysis [15, 16]. Molecular tools such as DNA microarrays [17, 18], RNA interference (RNAi) [10, 19], generation of transgenic lines [20, 21], and high throughput transcript sequencing approaches [22] are readily available. All these factors have contributed to make* A. aegypti* an excellent model for the study of JH biosynthesis.

## 2. JH Structures, Functions, and Mode of Action

JHs are lipophilic molecules commonly produced and released into the hemolymph by the CA, generally a pair of endocrine glands connected to the brain [24]. The naturally occurring JHs are a family of acyclic sesquiterpenoids primarily limited to insects. Eight different forms of JH have been identified. JH III is found in the majority of insects studied [2, 25], including* A. aegypti* [11, 25, 26]. Five JHs have been reported in* Lepidoptera*: JH 0, JH I, JH II, JH III, and 4-methyl JH I [27–30]. In addition,* Drosophila melanogaster* CA secretes a bis-epoxide JH III (JHB III) [31], as well as methyl farnesoate (MF) [32–36]. Recently, another bis-epoxide form of JH III, skipped bisepoxide (JHSB III), has been reported in heteropteran insects [37, 38]. At least one JH homologue has been identified in over 100 insect species covering more than 10 insect orders [2]. With more than 2.5 million insect species estimated to inhabit earth [39], it is reasonable to think that additional forms of JH might be discovered in the future.

The JHs are involved in reproduction, caste determination, behavior, stress response, diapause, and several polyphenisms [40]. Understanding the mode of action of JH at the molecular level has been a major challenge in insect biology. The recent discovery that the JH-resistance gene,* Methoprene-tolerant* (*Met*), plays a critical role in insect metamorphosis [41–43] has been followed by a rapid increase in our understanding of JH signaling. Met is a bHLH-PAS protein, characterized by a short stretch of basic amino acids followed by a HLH domain and two variably spaced PAS domains (A and B) [44, 45]. The idea that JH could be an activating ligand for Met was surprising because there were no examples of bHLH-PAS proteins working as hormone receptors that act as ligand-dependent transcription factors [43].

To form active transcription factors, functionally specialized bHLH-PAS proteins, such as Met, pair with a partner of their family. JH-dependent interaction between Met and its partner Taiman/SRC requires the hormone to be bound to a specific ligand-binding site. Met binds JH and its mimics with high affinity through a well-conserved hydrophobic pocket within its PAS-B domain [45]. In the absence of JH, Met is present as an inactive homodimer. Upon JH binding to the PAS-B domain, Met undergoes a conformational change that liberates Met from the homodimer complex and allows it to bind Taiman [43, 45–47]. By sensing JH and forming a ligand-dependent complex with a partner of its own bHLH-PAS family, Met establishes a unique class of intracellular hormone receptors. The complex recognizes JH-responsive elements (JHRE) in the promoter of genes containing canonical E box motifs [45, 48, 49].

In mosquitoes, JH acts via Met to regulate posteclosion development of the fat body and plays a dual role. Thousands of genes are active when the JH titer is low and then are suppressed by the rising JH; other genes appear specifically when the JH titer is high [50, 51]. Jindra et al. [43] identified some of the outstanding questions that remain unanswered after the characterization of the JH receptor. Among them I would like to highlight the following two: (1) what is the relationship among different JHs, different bHLH-PAS proteins, and diverse biological functions of JH in different systems? (2) Are different JH homologues acting in distinct ways through different complexes involving Met, Taiman, Cycle, or members of the nuclear receptor superfamily such as ultraspiracle? Recent studies in the heteropteran linden bug,* Pyrrhocoris apterus*, indicate that JH stimulates oogenesis through Met and Taiman but regulates gene expression in the gut through interactions of Met with the circadian proteins Clock and Cycle [52]; the latter bHLH-PAS protein has indeed been shown to bind Met in a JH-dependent manner [53]. More answers to these questions are sure to be provided in the next few years.

## 3. JH Biosynthetic Pathway

**Figure 1:**
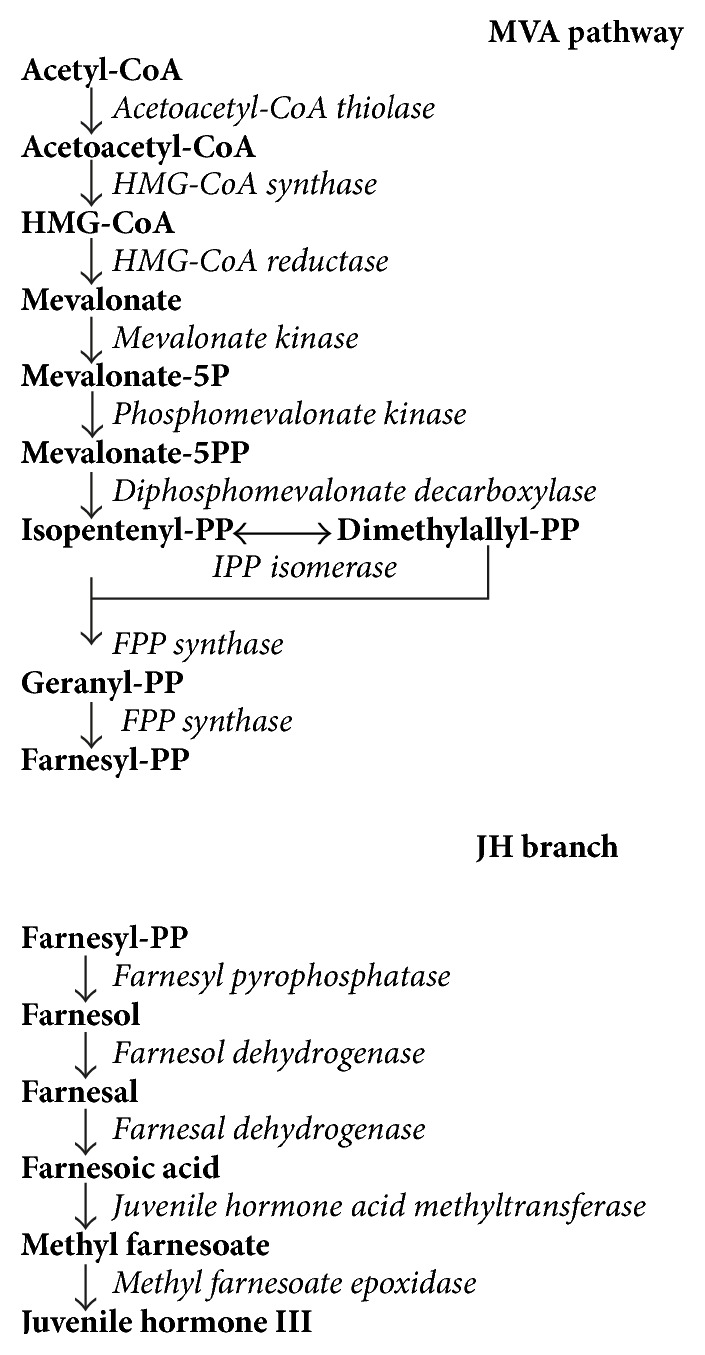
JH biosynthesis pathway. The biosynthesis of JH III involves 13 enzymatic reactions that can be conventionally divided into early (MVAP) and late (JH-branch) steps. Metabolites are shown in bold and enzymes in italic. Chemical structures are in [4].

JH is synthesized through the mevalonate pathway (MVAP), an ancient metabolic pathway present in the three domains of life [54]. The MVAP is responsible for the synthesis of many essential molecules required for cell signaling, membrane integrity, energy homeostasis, protein prenylation, and glycosylation [55–58]. The MVAP consists of a main trunk followed by subbranches that generate a diverse range of biomolecules. Insects lack the cholesterol-synthetic branch present in vertebrates, but in the CA the MVAP branches into the synthesis of JH [59]. The biosynthetic pathway of JH III in the CA of insects involves 13 discrete enzymatic reactions and is conventionally divided into early (MVAP) and late (JH-branch) steps [2] (Figure 1).

### 3.1. Early Steps (MVAP)

The early steps follow the MVAP to form farnesyl pyrophosphate (FPP) [59]. Initially, three units of acetyl-CoA are condensed into mevalonate by means of three sequential steps involving the enzymes acetoacetyl-CoA thiolase (THIOL), HMG-CoA synthase (HMGS), and HMG-CoA reductase (HMGR). Mevalonate is then converted to isopentenyl diphosphate (IPP) through three enzymatic reactions catalyzed by mevalonate kinase (MevK), phosphomevalonate kinase (P-MevK), and mevalonate diphosphate decarboxylase (PP-MevD) [6, 59]. FPP synthase (FPPS), a short-chain prenyltransferase, generates FPP by completing two sequential couplings: first IPP and dimethylallyl pyrophosphate (DMAPP) can condense in a head-to-tail manner to produce geranyl diphosphate (GPP). This type of head-to-tail condensation can be repeated by the further reaction of GPP with IPP yielding FPP.

FPP synthases have been identified from several insects and are typically active as homodimers [60–64]. In the mustard leaf beetle* Phaedon cochleariae*, FPPS possesses an interesting product regulation mechanism; it alters the chain length of its products depending on the cofactor present. The protein yields C_10_-GPP in the presence of Co^2+^ or Mn^2+^, whereas it produces the longer C_15_-FPP in the presence of Mg^2+^ [65]. That allows beetles to supply precursors for two terpene pathways, one for monoterpene metabolism (synthesis of chemical defenses) and one for sesquiterpene metabolism (JH formation), using only a single enzyme. The production of DMAPP, the allylic isomer of IPP, is catalyzed by an IPP isomerase (IPPI). Insect IPPIs require Mg^2+^ or Mn^2+^ for full catalytic activity [66, 67].

The enzymes of the MVAP are well conserved in eukaryotes; in insects all the MVAP enzymes seem to be encoded by single-copy genes, and identification of predicted amino acid sequences was possible based on sequence homology [4–6]. However biochemical characterization of purified or recombinant enzymes of the MVAP in insects is limited to HMGS [68], HMGR [69–71], IPPI [66, 67], and FPPS [60–65].

### 3.2. Late Steps (JH-Branch)

In the late steps of JH synthesis, conversion of FPP to farnesol (FOL) is catalyzed in* D. melanogaster* by a FPP phosphatase (FPPase or FPPP) [72], a member of the NagD halo alkanoic acid dehalogenase family (HAD), with orthologues in several insect species, including* A. aegypti* [73]. The mosquito FPPase (*Aa*FPPase-1) is a Mg^2+^-dependent NagD HAD protein that efficiently hydrolyzes FPP and GPP, but not IPP [73]. Afterwards farnesol undergoes two sequential oxidation reactions that generate farnesal and farnesoic acid (FA). In mosquitoes, the first reaction is catalyzed by a short chain farnesol dehydrogenase (*Aa*SDR-1), a member of the “classical” NADP-dependent cP2 SDR subfamily that presents broad substrate and tissue specificity [9]. Oxidation of farnesol into farnesal in mosquitoes is effected by a NAD^+^-dependent aldehyde dehydrogenase class 3 (*Aa*ALDH3-1) showing tissue and developmental-stage-specific splice variants [10]. Homologues of farnesol and farnesal dehydrogenases having similar activities in the CA of other insects have not yet been described.

The order of the last two biosynthetic steps, methyl esterification and epoxidation, catalyzed by a JH acid methyltransferase (JHAMT) and an epoxidase (EPOX), differs between insect species [2, 74]. In all insect species studied, recombinant JHAMTs were able to methylate JH III acid (JHA) and FA at similar rates [7, 75–79]. Homology modeling and docking simulations confirmed that JHAMT is a promiscuous enzyme capable of methylating FA and JHA [74]. In contrast, epoxidases have narrow substrate specificity; while the EPOX from the cockroach* Diploptera punctata* efficiently epoxidizes MF and is unable to process FA [80],* Bombyx mori* EPOX exhibits at least 18-fold higher activity for FA than MF [81]. Therefore, the order of the methylation/epoxidation reactions may be primarily imposed by the epoxidase's substrate specificity [74]. In* Lepidoptera*, epoxidase has higher affinity than JHAMT for FA, so epoxidation precedes methylation, while in many other insects there is no epoxidation of FA but esterification of FA to form MF, followed by epoxidation to JH III.

The late steps of JH biosynthesis were generally considered to be JH-specific [2] and the identification of these enzymes was hindered by the small size of the CA gland that made their isolation and biochemical characterization difficult. All the enzymes have now been characterized in insects using molecular approaches that included EST sequencing [4, 80], mRNA differential display [7], or homology to orthologue enzymes [10, 72]. Identification of the three enzymes involved in the conversion of FPP to farnesoic acid in mosquitoes has proven that the 3 proteins are encoded by families of paralogue genes with broad substrate specificity and expression in a wide number of tissues [9, 10, 78, 82]. This is not surprising since generation of farnesol by FPPase is important beyond the CA. Farnesol and farnesal homoeostasis are vital for cells in all insect tissues, and farnesol plays important roles in the regulation of a wide variety of cell functions, including proliferation and apoptosis [83–85], while posttranslational modifications by attachment of a farnesyl group to C-terminal cysteines of target proteins by farnesyl-transferases are essential for signal transduction and vesicular transport [86]. The presence of* Aa*FPPase,* Aa*SDR, and* Aa*ALDH3 isozymes with several isoforms capable of catalyzing each of the 3 enzymatic reactions in mosquitoes might have facilitated the evolution of more efficient substrate specificities, as well as a better tissue and developmental regulation. On the other hand, caution needs to be applied when trying to identify orthologues of these enzymes in other insect species, as not always the closest orthologue might play the same role in the CA.

On the contrary, the last two enzymes of the pathway (JHAMT and EPOX) are encoded by single genes in most insect species and are expressed predominantly in the CA [6, 7]. It is also noteworthy that EPOX genes appear to be insect-specific and have not been found in other arthropods. EPOX genes may be an evolutionary innovation that occurred in ancestral insects for the epoxidation of MF to JH [87].

### 3.3. Enzymatic Activities

The development of simple methods for detailed analysis of enzymatic activities derived from insect CA is critical. Fluorescence approaches are simplifying the study of the ability of CA extracts and recombinant enzymes to metabolize MVAP and JH-branch intermediates* in vitro* [11, 12, 73]. Eight selected enzymes have been evaluated using mosquito CA homogenates [12]. HMGS and JHAMT have the highest activities (in the nanomolar range), while the activities of additional six enzymes are in the femtomolar range (MK, PMK, FPPS, FPPase, farnesol dehydrogenase, and farnesal dehydrogenase).

## 4. Regulation of CA Activity

### 4.1. Mechanisms of Allatoregulatory Activity

Regulatory signals control the CA at least at three different levels [88, 89]. (1) Cytological/developmental responses are the gross morphological, microscopic, or enzymatic changes that determine the overall physiological status of the glands and their maximal potential output, for example, changes in cell volume and cell number which normally proceed in conjunction with developmental changes, such as the transition to adult [90]. (2) Constitutive/long-term responses, such as variations in enzyme levels during cycles of CA activity, are measured on a time scale of several hours to days. Examples of constitutive responses are the acquisition and loss of sensitivity to allatoregulatory peptides by the CA in* D. punctata* [91] and* A. aegypti* [92]. (3) Dynamic/short-term responses are measured on a time scale of minutes or hours and can be measured readily* in vitro*, such as the inhibition of JH synthesis by allatostatins or the stimulation of JH synthesis by allatotropin. These responses are usually reversible upon removal of the stimulus [93].

### 4.2. Nutritional Regulation of JH Synthesis and the Brain

The correct allocation of nutrients between competing needs such as reproduction, growth, maturation, or flight is a vital component of an insect's life-history strategy [94, 95]. Juvenile hormone has been described as part of a transduction system that assesses nutritional information and regulates reproduction in mosquitoes [96]. The nutrition-dependent development of the ovaries is an excellent physiological framework to understand the dynamic changes in JH biosynthesis during the gonotrophic cycle of female mosquitoes [12].

**Figure 2:**
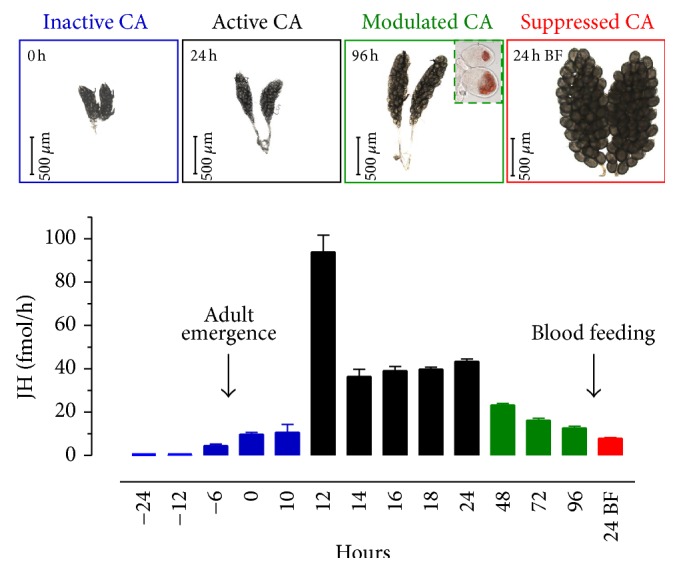
JH biosynthesis rates and ovarian development in female mosquitoes. Top panel: representative images of the progression of ovary development from emergence to 24 h after blood feeding. The inset in 96 h shows the lipid content of follicles from females fed 3% sugar (top) and 20% sugar (bottom). Colors for the panels match the colors for the nutrition-dependent physiological states of the CA shown in the panel below. Bottom panel: JH biosynthesis by CA dissected from pupa, sugar-fed, and blood-fed adult females. Hours represent times before (pupa) and after adult emergence (sugar-fed), or after blood feeding (BF). *Y*-axis: JH biosynthesis expressed as fmol/h. Bars represent the means ± SEM of three independent replicates of three groups of 3 CA. Colors represent the four distinct CA physiological phases identified: inactive or low activity CA (blue), active CA (black), modulated CA (green), and suppressed CA (red), from [12].

Three sources of nutrients provide energy and building blocks for the three distinct phases of ovarian development in* A. aegypti*. Preimaginal reserves are partially consumed during previtellogenesis (PVG); nectar-feeding adds reserves during the ovarian resting stage (ORS); and later a blood meal triggers vitellogenesis (VG) [96–101]. JH synthesis and ovarian previtellogenic maturation are activated in newly eclosed* A. aegypti* adult females only if teneral nutritional reserves are elevated [102]. Later, after previtellogenic maturation has been completed, JH mediates reproductive trade-offs in resting stage mosquitoes in response to nutrition [103]. Adult females* A. aegypti* show dynamic changes in JH biosynthesis, and regulation of the CA activity is quite different during previtellogenesis, the ovarian resting stage, and the vitellogenesis period [12] (Figure 2).

Comprehensive studies of transcripts, enzyme activities, and metabolites delimited four distinct nutrition-dependent CA physiological conditions that we named as follows: inactive, active, modulated, and suppressed CA (Figure 2) [12]. The molecular basis for JH synthesis regulation, as well as the role of brain factors or other endocrine regulators, might change during these 4 phases. We have previously described that transcript levels for most of the JH biosynthetic enzymes are very low in early pupae [6]; consequently JH synthesis rates were undetectable (below 0.5 fmol/h) in pupae 24 and 12 h before adult eclosion. Subsequently, in the last 6–8 h before adult emergence transcript levels for the biosynthetic enzymes commence to rise, the pupal CA becomes “competent” and starts to synthesize JH [6]. Although the CA of the newly emerged female is fully competent, for the next 10-11 h it synthesizes relatively low levels of JH (10 fmol/h) [12]. Decapitation during these first 12 h of imaginal life prevents increases of JH synthesis, suggesting that the brain plays a key role sensing the nutritional status and stimulating CA activity [104]. Only when preimaginal reserves are sufficient will the brain command the CA to synthesize enough JH to activate reproductive maturation [102].

Recent detailed studies in sugar-fed females revealed a previously undetected peak of maximum JH synthesis 12 h after adult emergence (Figure 2) [12]. This sharp increase in JH synthesis conveys information about teneral nutritional reserves and provides a signal to proceed with the previtellogenic maturation of the ovaries. The process of “activation” of CA is very fast and short lasting; JH synthesis increases from 10 fmol/h to almost 100 fmol/h in 2 h and decreases to less than 40 fmol/h in the next 2 h, remaining at this relatively high and constant rate until 24 h after emergence. Well-nourished females would activate the CA, increase JH synthesis levels, and complete the previtellogenic development by 48–60 h after emergence even if raised on water [104, 105].

If mosquitoes are nutritionally stressed, by 48–72 h JH synthesis is significantly reduced. This period represents the beginning of the ORS and female mosquitoes often ingest sugar meals to supplement their partially depleted preimaginal reserves. During the ORS, if nutrients are scarce, the brain directs the CA to “adjust” to the new adult nutritional condition; in mosquitoes fed a restricted diet such as 3% sugar, JH synthesis decreases to a low 12 fmol/h, triggering the resorption of ovarian follicles [95]. Decapitation during this ORS precludes this nutritional adjustment and causes significant increases in JH synthesis, emphasizing the critical role of the brain in CA nutritional modulation [104]. Finally, at 24 h after blood feeding there is an “active” suppression of JH synthesis that is critical for the completion of the vitellogenic development of the first batch of eggs and the triggering of the previtellogenic development of follicles for the second gonotrophic cycle (Figure 2) [12].

A coordinated expression of most JH biosynthetic enzymes has been previously described in mosquitoes and silkworms [6, 100, 101]. Increases or decreases in transcript levels for all the enzymes are generally concurrent with increases or decreases in JH synthesis [5, 6, 12], suggesting that transcriptional changes are at least partially responsible for the dynamic changes of JH biosynthesis. Most studies on JH synthesis have been performed using* corpora allata*-*corpora cardiaca* complexes (CA-CC). The 2 glands are very small and are intimately connected, so separating them is challenging. The synthesis of JH occurs exclusively in the CA; expression of the JH biosynthetic enzymes has been detected in the CA, but not in the CC of* B. mori* [106], and expression of the last 2 enzymes is also much higher in CA than CC in* A. aegypti* [107]. A potential role of the CC on CA regulation has been proposed in* B. mori* [108, 109]; separation of the CA from the CC often results in increases of JH synthesis* in vitro* in* A. aegypti* [93].

## 5. Allatoregulators

There are factors that can stimulate (allatotropins) or inhibit (allatostatins) CA activity [2]. In different insect species and at different stages of development, these regulatory factors may include three types of inhibitory allatostatins (AST), at least one type of stimulatory allatotropin (AT), insulin, and perhaps additional neuropeptides [110]. These factors were reviewed in detail in several recent articles [1, 2, 110–112].

### 5.1. Allatostatins and Allatotropins

Three families of allatostatins have been identified in insects: cockroach allatostatins (YXFGL-amide or type-A), cricket allatostatins (W2W9 or type-B), and* Manduca* allatostatins (PISCF or type-C) [111, 113, 114]. Each of the three structurally unrelated types of allatostatins (A, B, and C) is associated with a unique G-Protein-Coupled Receptor (GPCR) family that includes vertebrate orthologs. The AST-A receptors are related to the vertebrate galanin receptors [115], the AST-B receptors to the bombesin receptors [116], and the AST-C receptors show similarity to the somatostatin/opioid receptors [117, 118]. The AT receptor is also a GPCR and shows homology to the vertebrate orexin/hypocretin receptors [107, 108, 119, 120]. Stimulatory and inhibitory effects of brain factors have been described in mosquitoes [93, 104, 121]. Allatostatin-C and allatotropin are present in the brain of* A. aegypti*, [122]; they both modulate JH synthesis* in vitro* [92, 123] and their receptors are expressed in the CA-CC complex [107, 118]; however, their exact roles* in vivo* and mechanisms of action still need to be elucidated.

### 5.2. The Insulin/TOR Signaling Network

The insulin/TOR signaling network is evolutionarily conserved in most eukaryotes and plays a central role in the transduction of nutritional signals that regulate cell growth and metabolism [124, 125]. There are several reports suggesting that the insulin pathway modulates JH synthesis in insects. In* D. melanogaster,* specific silencing of the insulin receptor (InR) in the CA completely suppresses HMG-CoA reductase expression and renders a JH-deficient phenotype [126]. In addition,* D. melanogaster* InR mutants have reduced JH synthesis [127]. In* Culex pipiens*, the ability to enter into overwintering diapause is regulated by JH [128], and suppression of allatotropin simulates reproductive diapause [121]. In* C. pipiens,* silencing the InR or the downstream FOXO protein (forkhead transcription factor) by RNAi leads to a diapause phenotype [128]. The insulin/TOR pathway has also been suggested as a link between nutritional signals and JH synthesis regulation in the CA of the cockroach* Blattella germanica* [129, 130], and FOXO knockdown using systemic RNAi* in vivo* in starved females elicited an increase of JH biosynthesis [131].

The* A. aegypti* genome encodes eight insulin-like peptides (ILPs), with three of them (ILP1, ILP3, and ILP8) specifically expressed in brains of adult females [132]. ILP3 binds the* A. aegypti* insulin receptor (InR) with high affinity and has been described as a critical regulator of egg production [133]. Transcript levels for several* A. aegypti* ILPs show age-dependent and diet-dependent changes in female mosquitoes [134]. Mosquito ILPs action appears to be mediated by the tyrosine kinase activity of the mosquito insulin receptor and a signaling network involving phosphatidylinositol 3-kinase [135]. Selective activators and inhibitors of insulin signaling cascades had strong effects on insulin-regulated physiological processes in mosquitoes [135]; for example, knockdown of the* A. aegypti* phosphatase and tensin homolog (*Aaeg*PTEN) affects insulin signaling [136].

**Figure 3:**
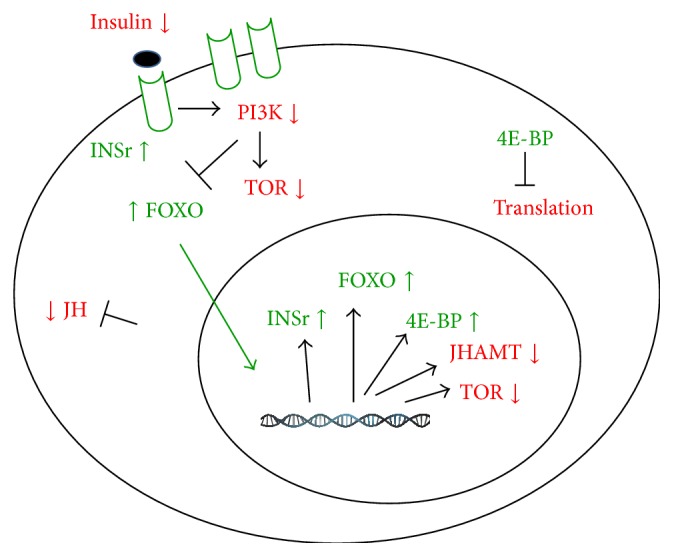
Starvation effects on insulin signaling components and JH synthesis in the CA of mosquitoes. This scheme summarizes starvation-related changes of insulin/TOR pathway components and JH synthesis. Molecules in red color are downregulated (↓), while those in green are upregulated (↑). Phosphoinositide 3-kinase (PI3K) and TOR are involved in the transduction of insulin signaling in the CA [13]. A starvation-dependent decrease of insulin results in an increase of FOXO signaling that promotes activation of transcription of insulin receptor (INSr) and 4E-binding protein (4EBP). Transcripts levels for FOXO increase and mRNAs for JHAMT and TOR decrease. JH synthesis decreases, while increases of 4EBP inhibit translation and increases of INSr enhance insulin sensitivity, from [23].

Application of bovine insulin on the mosquito CA-CC incubated* in vitro* caused a strong and fast stimulation on JH synthesis [19]. Little is known on exactly how insulin/TOR signaling affects the activity of the CA. Systemic depletion of TOR by RNAi and administration of the TOR modulator rapamycin had inhibitory effects on JH synthesis in mosquitoes, with both treatments causing reductions in JH biosynthetic enzyme transcript levels [19]. In* A. aegypti*, starvation decreases JH synthesis via a decrease in insulin signaling in the CA (Figure 3). Starvation-induced upregulation of the insulin receptor, increased CA insulin sensitivity and “primed” the gland to respond rapidly to increases in insulin levels. During this response to starvation, the synthetic potential of the CA remained unaffected, and the gland rapidly and efficiently responded to insulin stimulation by increasing JH synthesis to rates similar to those of CA from nonstarved females [23].

### 5.3. Additional Allatoregulatory Factors

Several additional factors have been proposed to be involved in the regulation of JH biosynthesis by the CA, including biogenic amines, 20-hydroxyecdysone (20E), ecdysis triggering hormone (ETH), and short neuropeptide F (sNPF) [2]. The steroid hormone 20E controls molting, metamorphosis, and oogenesis in insects [137–139]. 20E modulates JH synthesis in* Bombyx mori* larvae [140, 141], possibly by means of a direct control on the expression of some of the JH biosynthetic enzymes [109].

ETH is a small C-terminally amidated peptide, known as a major regulator of ecdysis in insects [142, 143]. Its role in inducing a stereotypical ecdysis behavioral sequence resulting in molts is well characterized [144]. ETH is synthesized and secreted into the hemolymph by specialized endocrine cells called Inka cells [142]. In* A. aegypti*, Inka cells are located along branch points of major epitracheal trunks [145]. The* A. aegypti* ETH gene encodes two isoforms of the 17 amino acid peptides, ETH1 (*Ae*ETH1) and ETH2 (*Ae*ETH2) [145]. Both of these peptides induce a receptor-mediated signaling cascade in CNS neurons that result in activation of motor programs allowing shedding of the old cuticle [142]. Yamanaka and collaborators reported very high expression of the ETH receptor in the CA of* B. mori* leading them to suggest that ETH might have a role in regulation of JH synthesis [108]. Preliminary results indicate a stimulatory effect of ETH on JH synthesis in* A. aegypti* during the maturation process of the CA in the last six hours before adult emergence, a time when genes encoding JH biosynthetic enzymes become transcriptionally active and the CA starts synthesizing basal levels of JH III [146].

The short neuropeptide F (sNPF), among other functions, modulates feeding, metabolism, reproduction, and stress responses in insects [147]. sNPF has been reported as an allatoregulatory peptide in* B. mori*; in the silk moth, the AT receptor is not expressed in the CA, but rather in the* corpora cardiaca* (CC), specifically in a group of 4 cells that express the sNPF [108]. According to the model proposed for* Bombyx*, AT inhibits the release of sNPF, and this peptide inhibits JH synthesis; so AT exerts an indirect allatotropic effect by “derepression.” This model has not been tested in mosquitoes or additional insect species.

**Figure 4:**
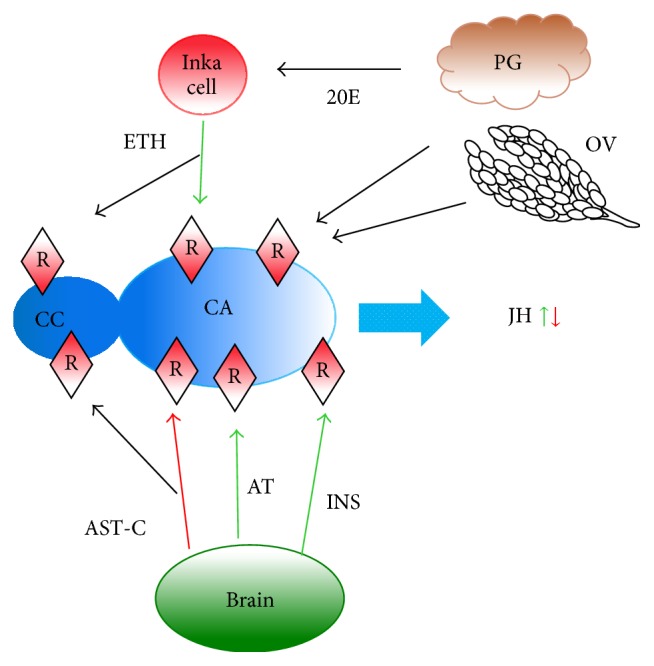
Effect of modulators on JH biosynthesis in female mosquitoes. Schematic representation of some of the tissues and molecules involved in JH biosynthesis regulation in mosquitoes. PG: prothoracic gland. OV: ovaries. CC:* corpora cardiaca*. CA:* corpora allata*. ETH: ecdysis triggering hormone. AST-C: allatostatin-C. AT: allatotropin. INS: insulin. 20E: 20 hydroxyecdysone. R: receptor. JH: juvenile hormone. Green arrow: stimulation. Red arrow: inhibition. Black arrow: modulation.

In mosquitoes, the role of each of these endocrine regulators might be limited to particular periods of CA activity. Developmental modulators such as ETH might play important roles during pupal maturation of the CA; insulin and/or allatotropin may well be the brain activators acting on the CA of the newly emerged female, while allatostatin-C and insulin could play a role in the nutritional modulation of JH synthesis during the “state of arrest,” as well as during the suppression of JH synthesis after a blood meal. In the CC-CA of mosquitoes, the expression of the following receptors has been detected: ETH A and B, ecdysone A and B, insulin, ultraspiracle A and B, allatotropin, AST-C A and B, and the short neuropeptide F. It is possible that signals from all these modulators are integrated in the CA, which suggests that the regulation of JH synthesis is extremely complex (Figure 4).

## 6. An Integrated View of Flux Control of JH Synthesis Rate

### 6.1. Flux Control

**Figure 5:**
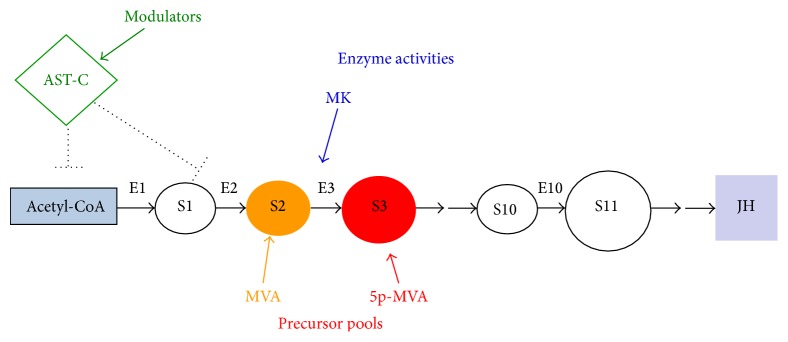
A schematic representation of a model for the control of the flux of precursors in the JH biosynthetic pathway. Precursor pools (S2, S3, etc.) are represented by circles and connected by arrows (MVA: mevalonic acid, 5P-MVA: mevalonate 5-phosphate). E: enzymes are followed by a number that refers to the position in the pathway (E3 = MK: mevalonate kinase). Regulatory factors might be affecting both precursor pool sizes and enzymatic activities (e.g., AST-C: allatostatin-C). JH: juvenile hormone, from [6].

JH synthesis is controlled by the rate of flux of isoprenoids, which is the outcome of a complex interplay of changes in precursor pools, enzyme levels, and external modulators such as nutrients and allatoregulatory factors [6, 12, 148, 149] (Figure 5). Discussion of the “control” or “regulation” of biosynthetic pathways normally focuses on the question of which individual enzymes are controlling the flux in a pathway [150, 151]. Flux is a systemic property, and questions of its control cannot be answered by looking at the different enzymatic steps in isolation. To understand how regulators modify JH synthesis, it is important to know their effect on the changes in the levels of all enzymes and precursor pool sizes.

The JH synthetic pathway involves 13 discrete enzymatic steps organized in an obligatory sequence. Each product represents the substrate for the next “downstream” enzyme. Enzymes are connected by metabolite pools that are common to them; for example, FOL is the product of the FPPase activity and the substrate for farnesol dehydrogenase. The pools are in fact the* links* in the system interactions; therefore, pool concentrations and fluxes (which are flows into and out of pools) are critical variables in JH regulation. The system's “*sensitivity*” to changes in the size of a precursor pool indicates the control importance of this enzymatic step in the final flux and can be experimentally tested. Although control of fluxes tends to be distributed among all enzymes in a pathway rather than confined to a single rate-limiting enzyme, the extent of control can differ widely between enzymes in a pathway [150]. It has been postulated that, in a synthetic pathway containing numerous enzymes, almost all the enzymes will appear to be “in excess,” in the sense that individual quantities or activities can be considerably reduced without appreciable effect on the flux [150]. Stimulation with exogenous precursors has been reported for the CA of many insect species, and it seems that having an excess of enzymes is common in most insects studied [6, 152–155]. In the CA of the cockroach* Diploptera punctata*, HMGS and HMGR activities are not always closely linked to the rate of spontaneous JH synthesis [154, 156]. Sutherland and Feyereisen [157] showed in* D. punctata* that inhibiting the HMGR activity by a third has a moderate inhibition of JH synthesis (less than 15%), indicating that this enzyme is in excess and has a low control coefficient on JH synthesis. Rate limiting bottlenecks have been proposed at single specific steps in both the MVAP and JH-branch in the CA of different insects, including upstream of the acetyl-CoA pool [157] as well as by rate limiting blockages at different enzymatic steps in the pathway, including the activities of HMGR [158, 159], farnesol dehydrogenase [9], farnesal dehydrogenase [10], or JHAMT [7, 77]. In contrast recent studies suggest that there are multiple regulatory points in the pathway and they might change in different physiological stages [12].

Branch point regulation is an important mechanism controlling carbon flow in the MVAP; the FPP produced by the MVAP can be shunted to many metabolic branches for the synthesis of critical molecules such as ubiquinone, dolichol, or prenylated proteins [59]. Remarkably, when the CA is very active, MVAP intermediate pools are completely depleted, implying that most MVAP precursors are channeled into the JH-branch. These results suggest that, during the peak of synthesis, the activity of the enzymes on the JH-branch is controlling the flux in the synthesis of JH, indicating that although CA cells are using the MVAP to synthesize additional metabolites that are important for various biological processes, when necessary, the production of JH supersedes the trafficking of FPP into other branches of the MVAP [12].

Compartmentalization of the enzymatic steps might add an additional level of complexity. Studies in plants have emphasized the importance of compartmentalization in the control of terpene biosynthesis [160], challenging the traditional view of isoprenoid metabolism occurring in a homogeneous environment with intermediates mixing freely and accessible to successive or competing enzymes. Experiments performed by Sutherland and Feyereisen [157] provided strong evidence that* D. punctata* CA glands inhibited with allatostatin-A (AST-A) were prevented from using glucose or amino acids to synthesize JH but free to utilize acetate; that is, AST-A was inhibiting steps in the glucose or amino acid (mitochondrial) incorporation pathway but not the acetate (cytoplasmic) incorporation pathway. Results from the* D. punctata*-AST-A model confirm that compartmentalization of the precursor pools and enzymatic steps is important and suggest that a major target of AST-A is either the transport of citrate across the mitochondrial membrane and/or the cleavage of citrate to yield cytoplasmic acetyl-CoA [157].

Metabolic enzymes that catalyze a series of successive reactions can form complexes on membranes or cytoskeletal structures [161]. Such metabolic enzyme complexes are called “metabolons,” functioning as metabolic channels that facilitate metabolite flux to committed end products [162]. Metabolons can “move” metabolites more efficiently through the pathway and limit the availability of potential common metabolite intermediates to other branches of the network [163]. Metabolon formation normally involves specific interactions between several “soluble” enzymes that might be anchored to a membrane either by membrane-bound structural proteins that serve as “nucleation” sites for metabolon formation or by membrane-bound proteins;* Aa*ADLH3 or epoxidase could serve that role in the CA of mosquitoes. In vertebrates, farnesal dehydrogenase closely interacts with farnesol dehydrogenase, forming a complex called “alcohol : NAD^+^ oxidoreductase” (FAO), responsible for the sequential oxidation of fatty alcohol to fatty acids [164, 165]. A similar complex including the two oxidoreductases, the JHAMT and epoxidase, might be present in the CA of mosquitoes, channeling precursors efficiently in the JH pathway.

*In vitro* experiments have shown that several intermediates in the pathway (e.g., mevalonate, farnesol, farnesal, and FA) are incorporated into the CA and stimulate JH synthesis [6, 25, 155]. It is puzzling that the CA of a newly emerged mosquito female that has a very large FA pool but limited JH synthesis is strongly stimulated by exogenous supply of FA [6, 12]. These results suggest differences in the channeling of “endogenous” and “exogenous” FA derived pools. In addition, there are examples of a reversal of the flux in the JH synthesis pathway, such as a reductase activity that converts FAL back into FOL in the CA of mosquitoes [10]. In the CA, some MVAP precursor pools might be controlled by feedback regulation imposed by metabolites such as FPP operating in the downstream portions of the pathway, in a similar mode to the negative feedback of late MVAP precursors (GPP, FPP) on the activity of mevalonate kinase described for terpene homeostasis in mammals [166].

**Figure 6:**
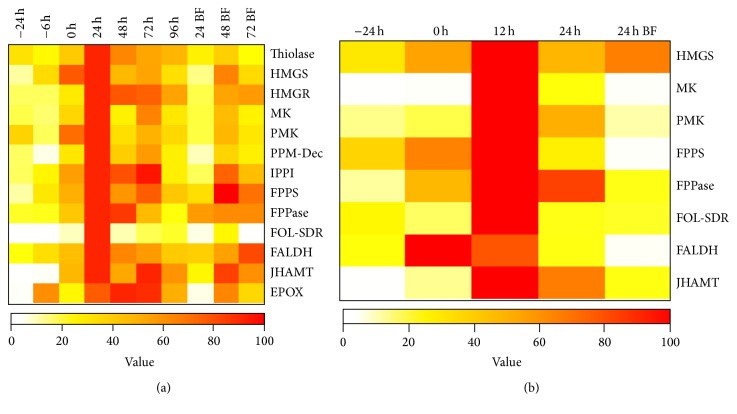
Heat map representation of changes in JH biosynthetic enzyme mRNAs and activities in CA extracts. (a) Changes in mRNAs encoding JH biosynthetic enzymes. (b) Changes in activities of JH biosynthetic enzymes in CA extracts. Top: physiological stages are described as hours relative to adult emergence (0 h) or blood feeding (BF). Right side: enzyme names abbreviations: Acetoacetyl-CoA thiolase: thiolase; HMG-CoA synthase: HMGS; HMG-CoA reductase: HMGR; mevalonate kinase: MK; phosphomevalonate kinase: PMK; diphosphomevalonate decarboxylase: PPM-Dec; IPP isomerase: IPPI; FPP synthase: FPPS; farnesyl pyrophosphatase: FPPase; farnesol dehydrogenase: FOL-SDR; farnesal dehydrogenase: FALDH; juvenile hormone acid methyltransferase: JHAMT; and methyl farnesoate epoxidase: EPOX. Colors from white to red represent increases of transcript levels or enzymatic activities as percentages of the maximum value, from [12].

**Figure 7:**
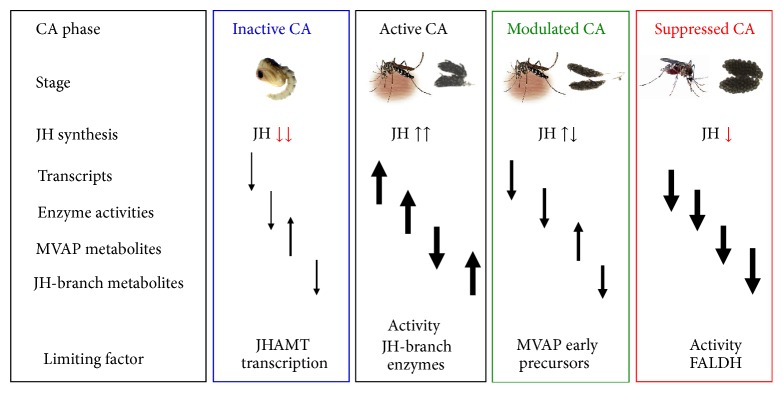
Schematic representation of the distinct four CA physiological conditions in reproductive female mosquitoes. The four CA phases and corresponding stages are as follows: inactive (early pupae), active (12–24 h sugar-fed females), modulated (48–96 h sugar-fed females), and suppressed (24 h blood-fed females). JH synthesis: the color and direction of the arrows reflect the following: low levels (arrows down and red), high levels (arrows up and black), or variable levels (arrow up and down). Changes in transcripts, activities, and metabolites are as follows: the direction of the arrows reflects the trend of the changes (increases: up and decreases: down); the size of the arrow reflects the magnitude of the changes, limiting factor: hypothetical critical factor limiting CA activity, from [12].

What do integrated studies of CA transcripts, enzyme activities, and metabolites tell us about the coordination of MVAP and JH-branch activities? A comprehensive analysis of the JH biosynthetic pathway has been done in* B. mori* [5, 106], showing that transcripts levels for the 8 enzymes of the MVAP and JHAMT are expressed in a highly coordinated manner during the 4th and 5th instar larvae as well as in pupae and adults. There is also a coordinated expression of the 13 JH biosynthetic enzymes in pupae and adults of female mosquito [6, 12]. The mosquito studies suggest that both pathways (MVAP and JH-branch) are transcriptionally coregulated as a single unit, and catalytic activities for the enzymes of the MVAP and JH-branch also change in a coordinated fashion in the “active” and “inactive” CA [12] (Figure 6). State-of-the-art metabolic studies were implemented for the first time to measure changes in all JH precursor metabolic pools in the CA of insects [12]. Unbiased Principal Component Analyses (PCA) showed that global fluctuations in the intermediate pool sizes in the MVAP and JH-branch are not functioning as a unit but behave inversely [12]. PCA of the metabolic pools changes indicated that, in reproductive female mosquitoes, there are at least 4 developmental switches that alter JH synthesis by modulating the flux at distinctive points in both pathways (Figure 7).

Further studies will be necessary to discover what enzymes restrict the flux into JH III at specific physiological conditions.

## 7. Challenges and Future Directions

JH has long been the focus of intensive research intended to exploit its properties for the purpose of generating novel pest control products. Earlier research on JH biosynthesis was performed mainly on three insect models: cockroaches, locusts, and moths. These insects offered several advantages for JH synthesis studies, such as the size of the CA, the relatively high levels of JH synthesized, and the easiness of rearing them in the laboratory. Cockroaches, in particular* D. punctata*, have been a favorite model because of many positive biological aspects, among them a clear correlation between cycles of JH synthesis and oocyte growth and vitellogenesis [167]. The moth* M. sexta* also provided an excellent endocrine system model amenable to the study of JH synthesis, in particular at the biochemical level, but did not offer the genetics necessary to further test many of the hypotheses generated by biochemical and physiological studies. The potential for genetic manipulation has made* Drosophila* the leader in the search for molecular mechanisms of action, but with the drawback of a lack of well-defined JH homologues and roles for JH biological activities. With the advent of genomic approaches, studies on other insect species such as* Tribolium* and* A. aegypti* are again contributing critical new insights into JH biosynthesis.

To answer the questions that remain unanswered, we need to identify some of the next challenges and future directions on JH synthesis research.

1. Although the general features of JH biosynthesis seem to be conserved in most insects, there is clearly diversity in aspects such as the presence of particular JH homologues, the order of the final enzymatic steps, and the role of allatoregulators; therefore JH biosynthesis studies need to be extended beyond the classic model insects.
2. The identification of all the genes encoding JH biosynthetic enzymes has allowed the completion of comprehensive transcriptional studies, as well as the expression and characterization of recombinant enzymes. New methods are currently facilitating the analysis of JH biosynthesis rates, enzymatic activities, and metabolite pool sizes in the CA. In the future, we should improve our understanding of the occurrence of different flux directionalities, feedback loops, and pathway branching points in the JH biosynthesis pathway.
3. More research on compartmentalization of JH synthesis is necessary, as well as a better understanding of signaling pathways in the CA, including calcium signaling pathways and the interactions among the insulin and TOR pathways.
4. The list of putative JH modulators continues to increase, and new concepts in allatoregulator-modulation of JH synthesis under different physiological frameworks are emerging.
5. The utilization of new statistical approaches, theoretical models, and system biology approaches should continue to simplify the interpretation of JH synthesis rates changes.

In summary, integrative approaches using CA metabolomics, genomics, and proteomics are promising tactics to identify regulatory points in the flux of precursors in the JH synthesis pathway and unveil the molecular mysteries of a complex metabolic system such as the synthesis of juvenile hormone in the* corpora allata* of insects.
